# Investigating the Role of Exercise in Improving Bone Health Among Elderly Women with Osteopenia

**DOI:** 10.3390/jfmk10040451

**Published:** 2025-11-20

**Authors:** Kyriaki Kotsili, Vasiliki Michou, Nikolaos Koutlianos, Anastasios Dalkiranis, Evangelia Kouidi, Asterios Deligiannis

**Affiliations:** Sports Medicine Laboratory, School of Physical Education and Sport Science, Aristotle University, 57001 Thessaloniki, Greece; kiriaki.kotsili2002@gmail.com (K.K.); vasilikimichou@yahoo.gr (V.M.); koutlian@phed.auth.gr (N.K.); tdalkiranis@phed.auth.gr (A.D.); kouidi@phed.auth.gr (E.K.)

**Keywords:** osteopenia, osteoporosis, exercise training, functional capacity, bone mineral density

## Abstract

**Background**: This study aimed to examine the impact of a 4-month multicomponent exercise program on bone and functional health in older women with osteopenia. **Methods**: Thirty women with osteopenia, aged 66.96 ± 5.71 years, were randomly assigned to two groups. The exercise group (Group A) participated in a combined exercise training program for 4 months, while the control group (Group B) remained untrained. All participants underwent bone density testing using DEXA, along with biochemical testing for bone metabolism and mineral exchange. This included measuring serum levels of calcium, phosphorus, vitamin D, alkaline phosphatase, parathyroid hormone, calcitonin, and estrogen. Functional capacity was assessed using various tests, including the 6 min distance (6MWD) test, the Timed Up and Go test (TUG), the 30 s Sit-to-Stand test (30 s-STS), and the Berg Balance Scale. **Results**: At the end of the study, repeated measures analysis showed a significant effect of time, group, and the interaction between time and group on the average scores of the 6MWD, TUG, 30 s-STS, and Berg Balance Scale for Group A. In terms of DEXA measurements, there were significant effects of time, group, and their interaction on average scores of Bone Mineral Density (BMD) and the right total hip T-score for Group A. Additionally, a statistically significant interaction between time and group was observed for lumbar spine BMD (*p* = 0.006). A significant group effect was also noted on the total left hip T-score (*p* = 0.033). **Conclusions**: A 4-month multicomponent exercise program can improve bone health and functional capacity in older women with osteopenia.

## 1. Introduction

Osteopenia is characterized by low bone mineral density (BMD) that does not reach the diagnostic threshold for osteoporosis. It is defined operationally by dual-energy X-ray absorptiometry (DEXA) as a T-score between −1.0 and −2.5 [1]. It is also considered a preclinical, at-risk condition that can precede the development of osteoporosis. In contrast, according to the World Health Organization (WHO), osteoporosis is defined as a metabolic bone disease characterized by decreased bone density, the deterioration of bone tissue, disrupted bone microarchitecture, and reduced bone strength [2]. These factors lead to increased bone fragility and a higher risk of fractures [3]. Approximately 200 million people worldwide have osteoporosis, and this number is expected to triple over the next three decades [4]. In 2019, approximately 8.14 million women and 6.11 million men aged 50 years or older experienced a hip fracture, which is the most serious consequence of osteoporosis [5]. Recent recordings show that globally, approximately 1 in 3 women and 1 in 5 men aged ≥50 years will sustain an osteoporotic fracture during their lifetime [6]. Hip fractures are associated with a mortality rate of 20% [4] within the year following the fracture and can lead to reduced mobility [7].

Women are at a higher risk of developing the disease, compared to men, due to lower BMD and an increased likelihood of fractures from falls, primarily caused by reduced estrogen levels during menopause. Additionally, other functional losses may occur, such as decreased strength, muscle mass, balance, and total functional capacity, all of which can further increase the risk of falls [4]. For older women with osteopenia or osteoporosis, fracture prevention should start with general lifestyle measures. These include maintaining a body mass index (BMI) greater than 20 kg/m^2^, avoiding smoking, and limiting alcohol intake to no more than one drink per day [8,9,10]. It is also crucial to mitigate fall risk through structured exercise programs that focus on balance, strength, resistance, flexibility, and endurance training. Multifactorial programs that identify modifiable risk factors and deliver tailored interventions are essential [11,12].

Exercise-based protocols play a vital role in addressing physical risk factors, managing pain, overcoming functional limitations, enhancing physical fitness, and improving quality of life [13]. Regular physical activity helps to preserve bone mass, increase muscle strength, and improve postural control. Notably, aerobic, moderate, or high-intensity resistance training, even without accompanying medication, can slow down BMD loss in postmenopausal women with osteopenia or osteoporosis [14,15]. Multicomponent interventions that combine resistance, balance, aerobic, and flexibility training offer a more comprehensive approach than traditional single-mode programs. These interventions are particularly effective for older women who are physically inactive. Research has shown that multicomponent training, especially when enhanced with dedicated flexibility exercises, can improve various aspects of health and fitness, including posture, flexibility, functional capacity, muscle strength and power, agility, and cardiorespiratory fitness. Additionally, these programs are often structured in socially engaging, group-based formats that promote long-term adherence to exercise [16,17,18].

More recently, attention has turned to how exercise structure, technology, and their interaction with pharmacological strategies can increase bone strength and reduce fall risk. Results from clinical trials in generally healthy, already active older adults indicates that low-intensity, simple home-based exercise programs may be insufficient to produce measurable improvements in lumbar spine or femoral neck BMD, while daily vitamin D_3_ supplementation (2000 IU) appears to yield only small, clinically uncertain benefits for hip and lumbar spine BMD, mainly in men, compared to women [19].

Digital and game-based technologies have further broadened exercise options for individuals with low bone mass. Exergame-based balance training, utilizing platforms such as the Nintendo Wii, has improved timed up-and-go (TUG) performance, Berg Balance Scale (BBS) scores, and fall-related self-efficacy compared to standard home exercises in women with osteoporosis, suggesting that interactive, gamified balance training can enhance dynamic stability and reduce fall risk [20]. Likewise, virtual reality exergaming (VRE) protocols that integrate strength and balance training in osteo-porotic postmenopausal women have been shown to refine neuromuscular control (earlier and stronger muscle activation, reduced hip-to-ankle activity ratios), decrease the fear of falling, and improve functional balance and quality of life [21].

Beyond commercial exergames, new interventions utilizing virtual reality, tele-exercise platforms, and AI-assisted monitoring aim to personalize exercise, track performance, and provide feedback, enhancing safety and effectiveness for female adults with or without osteoporosis [22,23]. A multicenter randomized controlled trial is evaluating a 6-month, moderate-to-high-intensity tele-exercise program for women at high risk of osteoporotic fractures, focusing on new vertebral fractures and falls as primary outcomes [24]. This highlights the growing interest in technology-supported exercise for preventing fractures and falls. As the field advances, these innovative exercise programs show promise in improving bone health, balance, and functional capacity in postmenopausal women with osteopenia.

Despite the growing body of research, several significant gaps still exist. First, most exercise trials have primarily focused on women with established osteoporosis or generally healthy older adults. However, postmenopausal women with DEXA-defined osteopenia, a large and high-risk group, are less frequently studied as a separate clinical population. Second, the effects of supervised multicomponent training on BMD in specific regions, such as the lumbar spine and both hips, are not well-characterized. Furthermore, the existing evidence is inconsistent regarding exercise modalities, doses, and durations [25]. Third, relatively few studies have concurrently evaluated skeletal outcomes and a comprehensive set of functional measures (i.e., strength and balance), limiting our understanding of how densitometric changes relate to clinically meaningful functional improvements in women with low bone mass. Finally, although technology-based and remotely delivered programs are promising for enhancing adherence, most available data derive from short-term interventions with small sample sizes, and their implications for standard, supervised, multicomponent exercise protocols remain unclear. Given the high prevalence of osteopenia/osteoporosis in this population and the favorable adherence profiles of multicomponent programs [17], clarifying their skeletal and functional effects is clinically important. Accordingly, this study aimed to evaluate the impact of a 4-month multicomponent exercise intervention on bone and functional health in postmenopausal women with DEXA-defined osteopenia. The study’s novelty lies in the concurrent, region-specific assessment of BMD and T-scores at the lumbar spine (L1–L4) and both hips, alongside a comprehensive battery of functional capacity and balance measures, which enables the examination of densitometric changes and their functional correlates. We hypothesized that the supervised multicomponent program would produce favorable improvements in BMD and T-scores, as well as in functional performance and balance, compared with control, and that functional gains would correlate positively with densitometric outcomes.

## 2. Materials and Methods

### 2.1. Participants

Initially, women with osteopenia were recruited from municipal open care centers for the elderly in the Prefecture of Central Macedonia, Katerini, Greece, and screened for eligibility. Participants’ inclusion criteria were female gender, age ≥ 60 years to 75 years, clinically diagnosed with osteopenia or osteoporosis, confirmed by a DEXA scan (T-score ≤ −1.0), and the ability to walk independently. Exclusion criteria included: non-confirmed DEXA osteopenia, previous involvement in an exercise training program within the past 6 months, history of hip or vertebral fracture within the past year, neurological, cardiorespiratory, or musculoskeletal conditions that may limit the patient’s participation in this study and would make exercise unsafe, and intellectual impairment or disorder that would prevent understanding instructions.

### 2.2. Sample Size Estimation

The required sample size was estimated based on a similar study by De Oliveira et al. [26] in the older postmenopausal women. We hypothesized statistically significant differences between groups (Group A vs. Group B) in BMD and indices of functional capacity and balance. Assuming a two-tailed α of 0.05 and 80% power, the required sample size was 14 participants per group. Allowing a 20% dropout rate, we aimed to recruit 30 participants (15 per group). A post hoc power analysis, conducted using a two-tailed test of significance, revealed that the trial had a power range of 71.9% to 72.8% when evaluating functional capacity [measured by the 6-Minute Walk Distance (6-MWD) and the 30-Second Sit-to-Stand test (30 s-STS)] as well as BMD. The analysis indicated that the required sample size for adequate power post hoc was 28 patients (14 in each group) by the end of the 4-month exercise training period.

### 2.3. Study Design

Initially, the detailed research protocol was submitted to and approved by the Research Ethics Committee of Aristotle University of Thessaloniki, receiving the relevant approval (protocol number: 241/2025). Following this, an invitation to participate in the study was announced at municipal open care centers for the elderly in the Prefecture of Central Macedonia, Katerini, Greece. This invitation included a thorough description of the study’s purpose, significance, and methodology. Women who volunteered to participate were asked to provide written informed consent, in accordance with the ethical principles outlined in the Declaration of Helsinki (2013). Then, women with osteopenia who met the inclusion criteria underwent a review of the medical history, a DEXA scan both in lumbar spine (L1–L4) and right and left hip for the radiographic diagnosis of osteoporosis, a 6-MWT, a TUG, a 30s- STS and a 8 Foot Up-and-Go (8-FUAG) test, to assess functional capacity, balance assessment using the BBS, evaluation of fall frequency and blood sampling for biochemical assessment of bone metabolism indices. Following baseline assessments, participants were randomly allocated (1:1) to the exercise intervention (Group A) or control (Group B) via https://randomizer.org/ (accessed on 2 April 2025). Group A followed a 4-month exercise training program three times per week, while Group B did not participate in any structured exercise program during the study period. The clinical trial started in April 2025 and ended in September 2025. Upon the conclusion of the four-month study, the same researchers, who were unaware of the group allocation, conducted all tests once more.

### 2.4. Functional Capacity, Balance and Fall Frequency Measurements

Exercise functional capacity was first assessed in both groups using the 6MWD, a validated measure in older adults [27,28]. Participants were instructed to wear comfortable clothing and footwear and to take their usual medications. The 30 m corridor course was measured with a trundle wheel, and the turnaround points were marked with tape. After standardized pre-test instructions and a 1 min warm-up, participants walked unassisted back and forth along the course for 6 min. Standardized, evenly toned encouragement was provided throughout. Test duration was monitored with a stopwatch, and the primary outcome 6MWD, in meters, was recorded on the data collection form.

Lower-limb functional strength was assessed with the 30 s-STS test, following the CDC STEADI protocol [29]. Participants sat in the middle of a standard armless chair (seat height ≈ approximately 45 cm/17 in), with their feet flat, back straight, and arms crossed at the wrists over their chest. On the examiner’s ‘Go’ command, they repeatedly rose to a full standing position and returned to sitting for 30 s. The examiner stood nearby for safety, used a stopwatch to time the trial, and counted the number of full stands completed; a rise that was >50% complete at 30 s was counted as a stand. If a participant used their arms to assist with standing, the test was terminated, and a score of 0 was recorded. Performance was expressed as total stands in 30 s, with lower scores indicating reduced strength/endurance. Age- and sex-specific thresholds below the average (screening for elevated fall risk) were applied according to reference norms. Focusing on older adults, 30 s-STS performance, indexed by total repetitions/time and derived power plus concentric/eccentric velocities, exhibited the strongest correlations with global function (SPPB) and handgrip strength in the >70-year subgroup (*p* < 0.01), underscoring 30 s-STS as a sensitive indicator of lower-limb function in late life [30].

The TUG test is a well-established, reliable measure of overall mobility and functional performance. For the test, participants sat in a 45 cm chair with their knees flexed to 90° and their hands crossed on their chest. On the examiner’s ‘go’ command, they stood up, walked 3 m, turned, and returned to the seated position. Execution time was measured with a stopwatch and recorded in seconds; shorter times indicate better performance. A threshold of 13.5 s is commonly used to identify individuals at increased risk of falls in community-dwelling populations [31,32].

The 8-FUAG test assesses dynamic balance. It involves getting up from a chair, walking as quickly as possible to a cone located 2.4 m (8 feet) away, going around the cone, and then returning to sit back in the chair. Completion time (seconds) was recorded, with shorter times indicating better performance [33]. The 8-FUAG test is a shorter version of the TUG test. Like the TUG test [31], it has demonstrated high test–retest reliability, with an Intraclass Correlation Coefficient (ICC) ranging from 0.92 to 0.97 (*p* < 0.001) [34].

The BBS is a 14-item assessment tool used by clinicians to evaluate balance in older adults. Each item is scored from 0 to 4, with a maximum possible score of 56. Scores lower than 56 indicate poorer balance, while scores below 45 suggest an increased risk of falls. The assessment requires minimal equipment, including two chairs, a step, a stopwatch, and a 4.6 m (15-foot) walkway. The BBS has demonstrated excellent reliability, with a Cronbach’s alpha of up to 0.97 in elderly populations [35]. In addition to the BBS item, fall frequency evaluation (mean number of falls) was also recorded before and after the end of the study.

### 2.5. Biochemical Markers of Bone Metabolism Assessment

Blood samples were collected after a 12 h fast. Blood analysis using biochemical auto-analyzer devices included the measurement of serum electrolytes (phosphorus), alkaline phosphatase (ALP), prolactin (PLR), parathyroid hormone (PTH), and 25-hydroxyvitamin D [25(OH)D].

### 2.6. Dual-Energy X-Ray Absorptiometry (DEXA)

BMD of the lumbar spine (L1–L4) and both the left and right total hip were measured using a DEXA scan (Hologic, Horizon DXA, Bedford, MA). BMD was reported as absolute values (grams per square centimeter) and T-scores, which indicate the number of standard deviations from the mean peak bone mass of healthy individuals of the same gender. BMD values were classified according to the World Health Organization guidelines established for postmenopausal white women: (a) Normal BMD: T-score greater than −1, (b) osteopenia: T-score between −1 and −2.5 (inclusive), (c) osteoporosis: T-score less than −2.5 [36].

### 2.7. Exercise Program

The intervention consisted of a supervised, multidimensional exercise program. The program included three sessions per week, each lasting 45 min, for a total of four months (approximately 16 weeks or 48 sessions). These sessions were conducted at a low-to-moderate intensity with a focus on progressive overload. The intensity was subjectively assessed using the Borg Scale of Perceived Exertion, likely with the 6–20 scale. Ratings of 11–13 were regarded as “light” to “somewhat hard”. Each session incorporated the following elements: (a) Lower-limb and trunk strengthening: Aimed at increasing muscle mass and reducing the risk of falls, (b). Balance and proprioceptive training: To enhance postural stability, (c). Floor-based Pilates: Adapted for older adults, with an emphasis on core control, posture, and breathing. The exercise program started with a 5 min warm-up of low-impact aerobics movements and active stretching of the upper and lower limbs and breathing exercises. The main part consisted of a 10 min aerobic training with cycling on a bicycle ergometer or walking on a treadmill and continued with 10 min floor and standing Pilates-based exercises, which included kneeling or seated leg lifts, seated leg raises with a fitball held in hands, ball presses, lateral step/leg drags to the side, balance drills with a fitball, seated arm presses with a ball, quadriceps/torso co-contraction, and abdominal activation with a fitball. Thereafter, they engaged in a range of 1 to 2 sets of strengthening exercises for the shoulder press, bicep curl, triceps extension, and leg flexion-extension, each requiring 8–12 repetitions. For this workout they followed a 30 s period between each exercise and progressively added the second set, lasting about 10 min. They utilized a commercial weight machine. The last part of each session consisted of 5 min of balance training and a 5 min cool-down.

Simple equipment, such as small balls and a Pilates ring, was used to engage various muscle groups, promote neuromuscular coordination, and improve flexibility and alignment. A qualified physical education instructor with experience working with older adults and special populations conducted the exercise training program in a municipal gym. Attendance was systematically recorded. Participants’ adherence and performance were monitored at each session, with exercise progressions or modifications made as needed to ensure safety.

### 2.8. Statistical Analysis

Between-group changes over time were examined using a two-way repeated-measures ANOVA. Assumptions for ANOVA were checked: normality of the main outcomes was assessed with the Shapiro–Wilk test and inspection of Q–Q plots, and homogeneity of variances was evaluated with Levene’s test. Both tests were non-significant (Shapiro–Wilk *p* = 0.12–0.89; Levene *p* = 0.21–0.77), indicating that model assumptions were satisfied for this sample. Effect sizes for ANOVA terms were quantified with partial eta squared (ηp^2^). Between-group differences at specific time points were evaluated with independent-samples t tests. Normally distributed variables are reported as mean ± standard deviation. Pearson’s correlation coefficients were computed to assess associations between functional capacity test scores, BMD, and total hip T-scores. Statistical significance was set at α = 0.05 (two-tailed). Analyses were performed in IBM SPSS Statistics, Version 27.0 (IBM Corp., Armonk, NY, USA; 2020).

## 3. Results

### 3.1. Participants’ Characteristics

**Figure 1:**
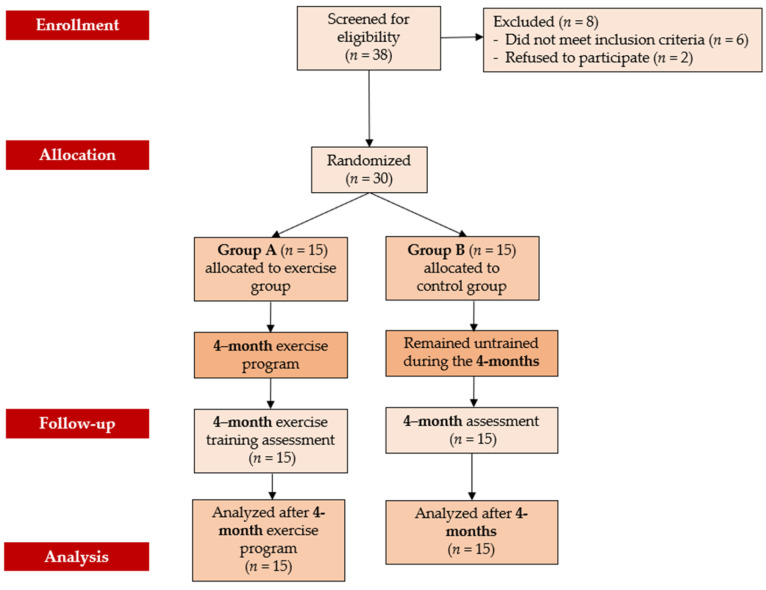
Flow chart consort diagram of the study.

Initially, 38 elderly women with osteopenia were screened for eligibility. Out of these, 30 women who met the inclusion criteria and volunteered to participate in the study were randomly assigned to either Group A (the exercise group) or Group B (the control group). All participants completed the study, with none withdrawing during the follow-up period, resulting in 30 women finishing the program. Group A demonstrated high adherence, with an attendance rate of 87% for scheduled sessions. Over the 4-month duration of the exercise program, which combined aerobic and resistance training, no musculoskeletal or cardiovascular complications related to the exercise were observed. Figure 1 presents the flow chart of the study, while Table 1 displays the baseline characteristics of the participants.

### 3.2. Functional Capacity, Balance and Fall Frequency Analysis

Repeated-measures ANOVA showed significant main effects of group, time, and group × time interaction for 6-MWD, TUG, 30 s-STS, and BBS (all *p* < 0.05), with medium-to-large effect sizes (ηp^2^ = 0.348–0.769). In Group A, these effects were reflected in favorable percentage changes with corresponding 95% confidence intervals, as summarized in Table 2. In contrast, no significant group effect was observed for 8-FUAG (*p* = 0.063, ηp^2^ = 0.226) or fall frequency (*p* = 0.115, ηp^2^ = 0.168), although fall frequency showed significant main effects of time (*p* = 0.022, ηp^2^ = 0.322) and group × time interaction (*p* = 0.016, ηp^2^ = 0.349) (Table 2).

### 3.3. Biochemical Markers of Bone Metabolism Analysis

In contrast, at the end of the 4 months, the repeated measures analysis revealed no statistically significant effect of time, group, or group × time interaction on any biochemical markers of bone metabolism for Group A (all *p* > 0.05; ηp^2^ = 0.000–0.209) (Table 3). The corresponding percentage changes and their 95% confidence intervals are reported in Table 3.

### 3.4. DEXA Measurements

Regarding the DEXA measurements, repeated-measures ANOVA showed significant effects of group, time, and group × time interaction on right total hip BMD and T-score (all *p* < 0.05; ηp^2^ = 0.501–0.725), which corresponded to the dominant limb. A significant group × time interaction was also observed for total left-hip BMD (*p* = 0.006, ηp^2^ = 0.434), and a significant group effect for the left total hip T-score (*p* = 0.033, ηp^2^ = 0.286). The corresponding mean percentage changes and their 95% confidence intervals for all BMD and T-score variables are presented in Table 4. In contrast, lumbar spine BMD and total spine T-scores did not show significant changes (all *p* > 0.05; ηp^2^ = 0.109–0.209) (Table 4).

### 3.5. Associations Between DEXA Results and Functional Performance

Since the exercise program primarily focused on enhancing mobility, strength, and balance, we analyzed the correlations between the most responsive functional tests and the skeletal site that demonstrated the most significant training effect (right total hip BMD/T-score). This approach enabled us to investigate whether improvements in hip bone health were linked to functional gains.

**Figure 2:**
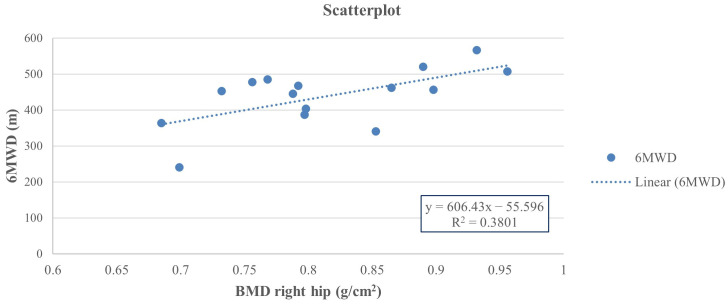
Pearson analysis between BMD of the right total hip (g/cm^2^) and 6MWD (m) (r = 0.617, *p* = 0.014) in group A.

**Figure 3:**
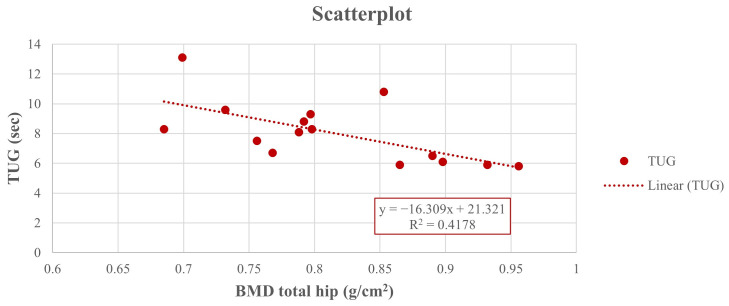
Pearson analysis between BMD of the right total hip (g/cm^2^) and TUG (sec) (r = −0.642, *p* = 0.001) in group A.

**Figure 4:**
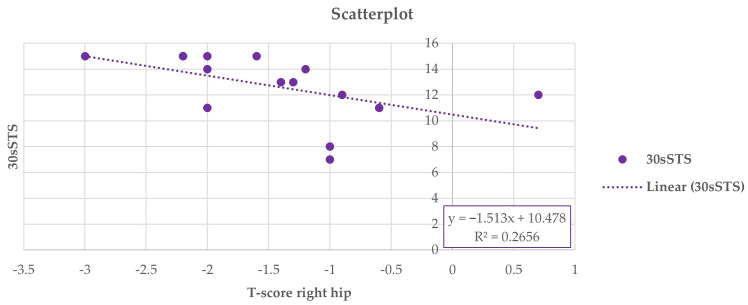
Pearson analysis between T-score of the right total hip and 30 s-STS (r = −0.515, *p* = 0.023) in group A.

Thus, at the end of the study, a positive Pearson correlation was found in group A between the 6-MWD score and right-total hip BMD (r = 0.617, 95% CI 0.222 to 0.876, *p* = 0.014), whereas a negative Pearson correlation was observed between the right-total hip BMD (Figure 2) and the TUG performance (r = −0.642, 95% CI −0.846 to −0.111, *p* = 0.001) (Figure 3) and between right total hip T-score and 30 s-STS performance (r = −0.515, 95% CI −0.861 to −0.164, *p* = 0.023) (Figure 4). Taken together, these associations suggest that women with better hip bone status also showed greater functional capacity (6MWD) and faster functional mobility (TUG), which are clinically relevant for fall and fracture prevention. The relationship between hip T-score and 30 s-STS further indicates that lower-limb muscular function is linked to hip bone characteristics in this population.

## 4. Discussion

Our study showed that a 4-month multicomponent exercise training program for older women diagnosed with osteopenia via DEXA was both feasible and effective. The program significantly enhanced bone health, as measured with BMD and T-score, along with improvements in functional capacity and balance. For individuals with osteopenia or osteoporosis, structured exercise can slow down or even partially reverse the decline in BMD [14,15]. This effect occurs because weight-bearing and resistance exercises create mechanical strain on the bones, which activates biological processes that promote bone health. Specifically, these activities enhance the activities of osteoblasts (cells responsible for bone formation) by increasing their proliferation and differentiation. Exercise also positively influences the RANKL-OPG pathway, which helps reduce the activity of osteoclasts, the cells that break down bone [37]. Furthermore, exercising improves blood flow to the bones, which assists in delivering nutrients and removing waste products. This shift in balance redirects remodeling dynamics toward bone formation, thereby improving microarchitectural integrity and bone mass [37].

Evidence suggests that exercise modality has site- and dose-specific effects on BMD, with varying efficacy across different protocols. The Italian Society for Osteoporosis, Mineral Metabolism and Bone Diseases recommends at least 30 min of daily physical activity (e.g., walking) for osteoporosis prevention and management [38]. However, systematic reviews suggest that walking alone is comparatively ineffective for preserving or increasing BMD, whereas progressive resistance training is consistently beneficial [39]. Modality–site specificity is also apparent: combined training appears most effective for lumbar-spine BMD, while resistance (strength) training exerts the most significant effects at the femoral neck [40]. Other studies report that resistance training improves femoral neck and lumbar-spine BMD but not whole-body BMD and identify whole-body vibration as a potential alternative for osteoporosis prevention [41]. Revisiting modality effects, Kemmler et al. [42] observed that weight-bearing interventions produced a more minor impact on lumbar-spine BMD than dynamic resistance programs. Yet, in further analyses, weight-bearing, dynamic resistance, and their combination did not differ significantly for lumbar spine, femoral neck, or total hip BMD, underscoring persistent uncertainty about the optimal prescription [42]. Overall, the most effective approach for maximizing BMD at specific skeletal sites remains unresolved [43]. Nevertheless, recent convergent evidence supports incorporating progressive exercise training into osteopenia or osteoporosis management as a versatile strategy that can meaningfully improve skeletal health and quality of life [44]. From the perspective of mechanical stimulation, resistance exercise applies a significant mechanical load to the bones, which stimulates and enhances the osteogenic response [45]. Research has also demonstrated that high-intensity resistance training performed twice a week or more yields the most substantial skeletal benefits [46]. Notably, a brief, supervised high-intensity resistance and impact training (HiRIT) program improved indices of bone strength and functional performance without adverse events in otherwise healthy postmenopausal women with low to very low bone mass, challenging assumptions about the safety and efficacy of higher-intensity protocols in this population [15]. However, these benefits may diminish if training is halted for more than six months [46].

Results from our study revealed that right total hip BMD in Group A increased by 6.57% over the 4-month multicomponent intervention, with significant main effects of group and time and a strong group × time interaction compared with Group B. A 6–7% increase in total hip BMD over just 4 months is likely to have significant clinical implications for postmenopausal women. Typically, this population experiences an age-related hip bone loss of about 1% per year. Therefore, reversing several years of expected loss in such a short time represents a meaningful change in the natural progression [47]. Furthermore, extensive prospective studies have demonstrated that hip and femoral neck BMD are strong, long-term predictors of hip and major osteoporotic fractures, extending up to 20–25 years. Women in the lowest BMD quartile face almost a four- to fivefold higher incidence of hip fractures over 25 years compared to those in the highest quartile [48]. A decrease in BMD of one standard deviation is associated with a two- to threefold increase in fracture risk [49]. Consequently, even modest gains in specific sites, especially at the hip, which is known for having the highest morbidity and mortality [50] associated with fractures, can lead to better long-term protection for skeletal health [51]. In this context, the hip BMD gains observed in the exercise group should be viewed as not merely statistically significant but potentially clinically meaningful, insofar as multicomponent exercise can address fracture risk through several converging pathways: (i) enhancement of balance and consequent reduction in fall risk, (ii) stabilization or augmentation of BMD at clinically relevant sites, and (iii) favorable alterations in bone structure [52]. Moreover, our study demonstrated that the exercised elderly showed a reduced fall frequency, which is of high clinical importance.

At the same time, the right-hip T-score improved in trained women, slightly declined in the controls, with significant effects of group, time, and group x time interaction. Moreover, the 4-month exercise training program led to a 2.7% increase in the left total hip BMD, with a significant group x time interaction (*p* = 0.006), whereas controls showed a slight decline. These results indicate that the right hip, being the dominant side and more involved in daily activities, has been determined to be more advantageous. In contrast, at the lumbar spine, neither BMD nor T-score showed significant main effects of time or group, nor a time-by-group interaction. Overall, our findings indicate that the hip-specific gains observed here are not only statistically significant but also clinically relevant, as they involve a fracture-critical site and coincide with improvements in mobility and balance that are likely to further attenuate fall-related fracture risk. However, currently, there is no definitive evidence that any specific exercise program can effectively reduce fracture risk, as no randomized controlled trial (RCT) has been sufficiently powered to demonstrate this outcome [52].

Furthermore, the above-mentioned results could be related to the content of the exercise program implemented in this study. According to the literature, the distinct effects of aerobic and resistance exercises on lumbar BMD may partly explain the difference in BMD between the lumbar and hip regions. However, combining resistance and aerobic exercise has been shown to effectively preserve lumbar BMD in postmenopausal women [53]. Other studies indicate that, at a microscopic level, the osteogenic index of a multicomponent exercise program is approximately double that of a single exercise program [54]. Moderate-intensity aerobic exercise can help protect bone and cartilage by regulating body trace elements that contribute to the formation of bone matrix structures. Additionally, it may inhibit bone resorption through an anti-free radical mechanism [55]. In contrast, to enhance hip BMD, it is recommended to engage in multidirectional exercises that incorporate odd-impact and moderate magnitude, creating unique loading directions. These exercises are linked to cortical thickening in the femoral neck, comparable to that resulting from HiRIT, making them a viable and targeted option for decreasing hip fragility [56]. However, in some studies, it is important to note that mixed-load exercise programs, which combine jogging with other low-impact activities, as well as resistance training programs that integrate impact activities with high-intensity exercises, appear to be effective in reducing postmenopausal bone loss and enhancing BMD in both the hip and spine [57].

Similar to our results, a systematic review and meta-analysis of six RCTs involving 391 participants (mean age 53–65 years) demonstrated that HiRIT led to greater improvements in lumbar spine BMD compared to moderate-intensity training, with minimal adverse events. However, there was significant variability in the studies included, and the overall certainty of the evidence was very low due to issues such as bias, inconsistency, and imprecision. The authors also suggested that although HiRIT may improve lumbar spine BMD in postmenopausal women with osteoporosis, these results should be interpreted with caution until higher-quality trials are conducted [58]. In addition, a previous review by Hoke et al. [59] highlights that, among exercise modalities for postmenopausal women with osteoporosis, resistance (weight-bearing) training has the most consistent, convincing impact on improving BMD, whereas aerobic, stretching, and balance-focused activities (including Tai Chi, Yoga, and Pilates) primarily help reduce fall risk by enhancing posture, core strength, and balance. Since preventing fractures depends on both increasing bone strength and reducing the risk of falls, exercise should be a fundamental aspect of comprehensive care, with a focus on long-term, sustained participation. A recent meta-analysis of 49 trials comparing eight different exercise modalities in postmenopausal women found that exercise interventions improved or reduced the loss of BMD at important skeletal sites [60]. For the lumbar spine, combined aerobic and resistance training was the most effective, followed by aerobic exercise and resistance training conducted separately. At the femoral neck, aerobic and resistance training again showed the best results, with whole-body vibration (WBV) and resistance training also proving to be effective. However, De Oliveira et al. [26] found that three weekly sessions of WBV or Pilates over six months (a total of 78 sessions) resulted in comparable and clinically significant improvements in BMD at both the lumbar spine and trochanter, compared to the control group. These results showed large effect sizes in postmenopausal women. Follow-up was completed by 96.1% of participants, and both WBV and Pilates demonstrated similar improvements in lumbar spine and trochanter BMD, with neither intervention showing statistical superiority over the other. No significant differences were observed at other skeletal sites between the two groups [26]. Overall, structured exercise, especially aerobic and resistance training, was considered the most effective approach for enhancing BMD in the lumbar spine and femoral neck in postmenopausal women [60].

Additionally, our study showed significant intra-group and inter-group changes over time. At the end of the exercise training program, Group A statistically increased average scores of 6MWD, TUG, 30 s-STS, 8-FUAG and BBS, compared to Group B, while significant effects of time, group and group × time interaction were also noticed. Our results were somewhat similar to those of the study by Wen et al. [61], which involved 48 postmenopausal women with osteopenia (lumbar spine BMD T-score of −2.00 ± 0.67). The participants engaged in a 10-week progressive, group-based step aerobics program (three times per week, 90 min per session, at 75–85% of heart rate reserve). This program resulted in a significant reduction in bone resorption, although there were no notable changes in osteocalcin or BMD after the end of the exercise intervention. Functional fitness improved markedly in the exercise group, with significant enhancements observed in chair stands, arm curls, the 8-FUAG, sit-and-reach, and the 2 min step test (*p* < 0.05). In contrast, the changes in the control group were not significant. These findings suggest that short-term step aerobics exercise favorably modulates bone turnover by reducing resorption while also enhancing physical function in postmenopausal women with osteopenia [61]. Likewise, Bragonzoni et al. [62], found that a 6-month multicomponent exercise program in postmenopausal women with osteoporosis can significantly enhance functional capacity, as determined by 6MWD, while Filipović et al. [63] observed [64] statistically significant improvements in TUG, STS, one-leg stance test and Fall Efficacy Scale (FES-I), after 4 and 12 weeks of exercise, respectively. Moreover, Otero et al. [65] showed that a long-term balance and strength training in older osteoporotic women had significant improvements in static balance by 21%, dynamic balance, as estimated using the 8-FUAG by 36%, and in the strength of the upper, as determined by arm curl test by 80% and lower limbs, as measured using 30 s-STS, by 47%. Research evidence supports that lower scores in the BBS [64], TUG [66], and other functional tests are recognized as strong predictors of falls. Moreover, research on group-based fitness interventions, such as dance programs for older adults, indicates that improvements in BBS scores are associated with significant reductions in falls and fall-related injuries [67]. This finding supports the use of the BBS not only as a clinical assessment tool but also as a reliable indicator of functional ability in fitness and physical activity programs aimed at older populations [67]. Since falls are a major cause of many osteoporotic fractures, the functional improvements observed in this study are likely to have significant clinical implications, particularly when they coincide with specific enhancements in hip BMD.

Also, by the end of the intervention, women in the exercise group showed a lower fall frequency compared with their baseline values. These results align with meta-analytic evidence from community-dwelling older adults, which shows that exercise, particularly balance and functional training, whether done alone or combined with resistance training, can reduce the risk of falls and major osteoporotic fractures by approximately 20–30% compared to usual care [68,69,70]. However, current evidence does not yet establish a single “optimal” exercise mode or dosage for fracture prevention, as few trials have been specifically designed to measure fracture outcomes, and significant methodological variability remains among studies [52]. Therefore, our findings support the incorporation of supervised, multicomponent exercise into the management of osteoporosis and osteopenia. However, falls are common among older women who have osteopenia or osteoporosis [1,25]. A possible explanation for this phenomenon is outlined in the study by Ishikawa et al. [71]. The study found that the loss of angulation in the curvature of the lumbar lordosis, which is a typical change in older women, increases postural instability. This instability increases the risk of falls due to the displacement of the gravitational line on the sagittal plane [71]. Multicomponent exercise programs effectively address the multifaceted nature of fall risk [72]. By combining strength training, balance exercises, flexibility work, and endurance activities, these programs enable older adults to improve various aspects of fitness [73]. Interestingly, a multicomponent exercise training, lasting about 27 weeks, with an average of 2.6 sessions per week for 45 min per session, was shown to improve balance, functional fitness, strength, flexibility, and BMD, thus reducing the risk of falls in older women with osteoporosis [4]. They can be tailored to individual abilities, ensuring safety and accessibility for different fitness levels. Research indicates that regular participation in these programs reduces fall risk by enhancing postural control, motor coordination, and responses to disturbances, all of which are vital for maintaining stability and mobility in later life [73,74]. Recent studies also emphasize the significant impact of WBV on reducing falls in this vulnerable population [60,75], even though WBV exercise regimens are highly controversial [75].

In contrast to the significant improvements observed in total hip BMD and functional outcomes, the biochemical markers of bone metabolism did not show differences between the groups after 4 months. This finding can be attributed to several methodological and physiological factors. Firstly, the intervention period was relatively short for serum markers, which exhibit substantial day-to-day and seasonal variability. Consequently, small exercise-induced changes may have been masked within the 4-month timeframe. Secondly, the multicomponent program was intentionally prescribed at a low-to-moderate intensity to ensure safety for women with low bone mass. As a result, the mechanical and osteogenic stimulus may not have reached the threshold necessary to produce a detectable systemic change in circulation. Conversely, studies that combined a higher exercise dose with or without supplementation have reported more pronounced skeletal effects. For instance, Alghadir et al. [76] found that 16 weeks of high-intensity interval training (HIIT) plus vitamin D led to greater BMD gains in osteoporotic women compared to each intervention alone. Additionally, Roghani et al. [77] observed an increase in bone-specific ALP after 18 sessions of progressively intensified submaximal treadmill walking (three times a week for six weeks). This suggests that both exercise intensity and progression can influence the biochemical response. The primary adaptations observed in our study were likely direct, site-specific responses of bone to weight-bearing and muscle-driven loading. These adaptations were mediated through mechanotransduction pathways, piezoelectric effects, and improvements in bone blood flow induced by exercise, which enhance local metabolism [78,79]. Such local skeletal changes do not necessarily result in measurable changes in systemic markers over a short period, mainly when the exercise load is divided among several components (such as strength training, balance exercises, and Pilates) rather than focusing solely on high-impact or high-intensity resistance training. Additionally, aerobic and weight-bearing exercises are believed to promote osteoblast enzyme activation [39]. However, this effect may require either a longer duration of exercise or a more targeted loading protocol to become evident in serum biomarkers. Therefore, the lack of significant biochemical change observed in the present trial should be interpreted as a limitation in marker sensitivity and intervention dosage, rather than evidence against exercise-induced bone adaptation.

We also found significant correlations between right total hip BMD and the 6-MWD score (*p* = 0.014), the TUG test (*p* = 0.001), and the right total hip T-score with the 30 s-STS performance (*p* = 0.023). These significant correlations support our functional findings, suggesting that improvements in mobility and lower-limb function may accompany, or at least co-exist with, better hip bone characteristics in postmenopausal women with low bone mass. In agreement with our results, Lindsey et al. [80] observed that among 116 healthy postmenopausal women, multiple physical performance metrics, including normal and brisk gait speed, normal and brisk step length, one-leg stance time, and grip strength, showed significant positive correlations with BMD across various skeletal sites (*p* < 0.05). These metrics also remained independently associated with site-specific BMD in multivariable models (adjusted R^2^ ≈ 0.11–0.24; *p* < 0.05). In contrast, the STS did not show any relationship with BMD at any site. While Moradell et al. [81] found a positive correlation between balance, trochanter BMD, and total hip BMD (r = 0.253 and r = 0.267, respectively; both *p* < 0.05) in older postmenopausal women. Meanwhile, Dai et al. [82] noted a positive relationship between the Short Physical Performance Battery (SPPB) total score, femoral neck BMD, and total hip BMD. Based on the above, it is clear that numerous studies have investigated the effects of exercise on functional capacity and bone mineral density BMD at the hip (including total hip and femoral neck) and lumbar spine in healthy older women. However, there is a lack of research specifically focused on postmenopausal women with osteopenia or osteoporosis that clearly tests and reports significant or non-significant associations. Our findings contribute valuable insights into this underexplored area.

To sum up, this study has both strengths and limitations. Firstly, this study found that a 4-month multicomponent exercise program had significant effects on BMD and functional capacity, but not on biochemical markers of bone metabolism, indicating that these effects were only attributed to exercise training. Secondly, it combined region-specific DEXA outcomes (lumbar spine; left/right total hip), a broad functional battery (6MWD, TUG, 30 s-STS, 8-FUAG, BBS), and biochemical profiling, enabling triangulation across skeletal, functional, and clinical domains. Thirdly, the program yielded significant gains in right- and left-hip BMD/T-scores and functional outcomes, and identified meaningful bone–function correlations, enhancing interpretability for practice. In contrast, modest sample size (n = 30) with single-center recruitment, a relatively short intervention (4 months) that may be insufficient to detect spine-specific densitometric change, absence of long-term follow-up, and limited control for potential confounders (e.g., habitual diet/supplement use, physical activity outside sessions) may be considered limitations of our study. Although we used two-way repeated-measures ANOVA, future studies with larger samples and more measurement occasions could profit from linear mixed-effects models, which flexibly incorporate random effects and are less sensitive to missing data. Lastly, findings in older Greek women with osteopenia may constrain generalizability to other settings and to women with established osteoporosis.

## 5. Conclusions

The present study found significant effects of a multicomponent exercise training program on bone health, as indicated by improvements in BMD and T-scores, as well as in functional capacity. Future research should involve larger multicenter trials with blinded outcome assessments and a minimum follow-up of 12 months. This approach will help evaluate the long-term benefits of hip gains, potential changes in the spine, and the incidence of falls and fractures as clinical endpoints. Additionally, comparative effectiveness studies should be conducted to contrast multicomponent training with higher-intensity resistance or impact training, as well as multidirectional loading. Dose–response analyses that account for factors such as frequency, velocity, and progression of training are also needed. Trials should stratify participants based on factors such as osteopenia versus osteoporosis, hip dominance, and baseline functional capacity. It is essential to incorporate standardized nutritional guidelines (calcium and vitamin D), device-based activity monitoring, and adherence and implementation outcomes (such as cost, scalability, and tele-supervision). Furthermore, mechanistic studies using bone turnover markers and advanced imaging techniques could help clarify site-specific adaptations and link changes in muscle power to microarchitectural remodeling of bone.

## Figures and Tables

**Table 1:** Clinical characteristics.

	Group A (n_A_ = 15)	Group B (n_B_ = 15)	A vs. B Group *p*-Value
Age (years)	67.86 ± 5.30	66.73 ± 5.18	*p* = 0.346
Height (cm)	158.80 ± 5.95	159.10 ± 4.67	*p* = 0.445
Weight (kg)	67.93 ± 13.98	69.13 ± 8.32	*p* = 0.423
BMI (kg/m^2^)	26.98 ± 5.67	27.42 ± 4.03	*p* = 0.398
Comorbidities (n, %)			
Hypertension	6 (40.0%)	5 (33.3%)	*p *= 0.476
Hyperlipidemia	4 (26.6%)	2 (13.3%)	*p *= 0.290

Note: BMI: Body Mass Index.

**Table 2:** Functional capacity, balance and fall frequency analysis of women at baseline and the end of the study.

	Group A (n_A_ = 15)			Group B (n_B_ = 15)			Group A vs. B		Analysis of Variance, *p* Value, ηp^2^		
	Baseline	Follow-Up	Change Mean (%), [95% CI: Lower/Higher Bound]	Baseline	Follow-Up	Change Mean (%), [95% CI: Lower/Higher Bound]	Change Mean (%), [95% CI: Lower/Higher Bound]		Group	Time	Group × Time
							Pre	Post			
6-MWD (m)	362.16 ± 61.09	437.99 ± 80.94	+75.83 (20.90%), [95% CI: 60.67, 90.96]	361.56 ± 70.28	361.03 ± 70.44	−0.53 (−0.15%), [95% CI: −0.95, −0.11]	−0.60 (−0.17%) [95% CI: −47.72, 46.52]	−76.96 (−21.31%) [95% CI: −131.25, −22.66]	F(1,14) = 19.348, *p* < 0.001 *, ηp^2^ = 0.580	F(1,14) = 23.964, *p* < 0.001 *, ηp^2^ = 0.628	F(1,14) = 36.974, *p* < 0.001 *, ηp^2^ = 0.701
TUG (s)	8.81 ± 2.30	8.04 ± 2.07	−0.77 (8.70%), [95% CI: −1.14, −0.37]	8.78 ± 2.33	8.80 ± 2.36	+0.02 (+0.23%), [95% CI: −0.06, 0.03]	−0.03 (−0.34%) [95% CI: −1.68, 1.62]	+0.76 (+8.64%) [95% CI: −0.82, 2.34]	F(1,14) = 7.488, *p* = 0.016 *, ηp^2^ = 0.348	F(1,14) = 14.025, *p* = 0.002 *, ηp^2^ = 0.500	F(1,14) = 15.727, *p* = 0.001 *, ηp^2^ = 0.529
30 s-STS (number of repetitions)	10.80 ± 2.67	12.66 ± 2.55	+1.86 (17.20%), [95% CI: 1.32, 2.39]	10.66 ± 2.25	10.53 ± 2.32	−0.13 (−1.20%), [95% CI: −0.30, 0.04]	−0.14 (−1.31%) [95% CI: −1.90, 1.62]	−2.13 (−20.23%) [95% CI: −3.87, −0.38]	F(1,14) = 17.707, *p* < 0.001 *, ηp^2^ = 0.558	F(1,14) = 37.260, *p* < 0.001 *, ηp^2^ = 0.727	F(1,14) = 46.667, *p* < 0.001 *, ηp^2^ = 0.769
8-FUAG (s)	8.32 ± 2.11	7.76 ± 1.92	−0.56 (6.70%), %), [95% CI: −0.83, −0.28]	8.28 ± 2.01	8.29 ± 2.03	+0.01 (+0.12%), [95% CI: −0.02, 0.03]	−0.04 (−0.48%) [95% CI: −1.51, 1.43]	+0.53 (+6.39%) [95% CI: −0.88, 1.94]	F(1,14) = 4.084, *p* = 0.063, ηp^2^ = 0.226	F(1,14) = 14.689, *p* = 0.002 *, ηp^2^ = 0.512	F(1,14) = 15.634, *p* = 0.001 *, ηp^2^ = 0.528
BBS score	51.13 ± 4.68	52.46 ± 3.70	+1.33 (2.60%), [95% CI: 0.62, 2.03]	50.86 ± 4.62	50.82 ± 4.73	−0.04 (−0.08%), [95% CI: −0.18, 0.06]	−0.27 (−0.53%) [95% CI: −3.59, 3.05]	−1.64 (−3.23%) [95% CI: −4.67, 1.39]	F(1,14) = 19.176, *p* < 0.001 *, ηp^2^ = 0.578	F(1,14) = 13.513, *p* = 0.002 *, ηp^2^ = 0.491	F(1,14) = 13.025, *p* = 0.003 *, ηp^2^ = 0.482
Frequency of falls (mean number of falls)	1.20 ± 1.09	0.26 ± 0.19	−0.94 (78.30%), [95% CI: −1.60, −0.25]	1.26 ± 1.14	1.33 ± 1.28	+0.07 (+5.60%), [95% CI: −0.06, 0.18]	+0.06 (+4.76%) [95% CI: −0.73, 0.85]	+1.07 (+80.45%) [95% CI: 0.41, 1.72]	F(1,14) = 2.818, *p* = 0.115, ηp^2^ = 0.168	F(1,14) = 6.646, *p* = 0.022 *, ηp^2^ = 0.322	F(1,14) = 7.500, *p* = 0.016 *, ηp^2^ = 0.349

Note: Data are expressed as mean ± SD; analysis of variance was expressed in both time, group and time × group as F[IV degrees of freedom (df), error df] = F-ratio, *p* = significant level, ηp^2^ = partial eta squared. * *p* < 0.05 indicates statistical significance. 95% CI: 95% confidence interval (lower bound/upper bound); Change values are Post—Baseline. Negative changes in time-based tests (TUG, 8-FUAG) indicate improvement. 6-MWD: 6-Min Walking Distance; TUG: Timed Up and go Test; 30 s-STS: 30 s Sit-To-Stand test; 8-FUAG: 8 Foot Up-and-Go Test; BBS: Berg Balance Scale.

**Table 3:** Biochemical markers of bone metabolism of the participants at baseline and the end of the study.

	Group A (n_A_ = 15)			Group B (n_B_ = 15)			Group A vs. B		Analysis of Variance, *p* Value, ηp^2^		
	Baseline	Follow-Up	Change Mean (%), [95% CI: Lower/Higher Bound]	Baseline	Follow-Up	Change Mean (%), [95% CI: Lower/Higher Bound]	Change Mean (%), [95% CI: Lower/Higher Bound]		Group	Time	Group × Time
							Pre	Post			
Vitamin D (ng/mL)	30.79 ± 10.55	30.68 ± 9.26	−0.11 (−0.41%), [95% CI: −3.63, 3.41]	30.62 ± 10.49	30.63 ± 10.58	+0.01 (+0.03%), [95% CI: −0.18, 0.20]	−0.17 (−0.56%) [95% CI: −7.69, 7.35]	−0.05 (−0.16%) [95% CI: −7.16, 7.06]	F(1,14) = 0.014, *p* = 0.908, ηp^2^ = 0.001	F(1,14) = 0.004, *p* = 0.953, ηp^2^ = 0.000	F(1,14) = 0.005, *p* = 0.943, ηp^2^ = 0.000
PLR (ng/mL)	10.14 ± 9.64	9.56 ± 3.28	−0.58 (−5.70%), [95% CI: −5.05, 3.89]	10.13 ± 9.65	10.12 ± 9.66	−0.01 (−0.10%), [95% CI: −0.03, 0.01]	−0.01 (−0.10%) [95% CI: −6.91, 6.89]	+0.59 (+5.83%) [95% CI: −4.60, 5.72]	F(1,14) = 0.059, *p* = 0.812, ηp^2^ = 0.004	F(1,14) = 0.068, *p* = 0.799, ηp^2^ = 0.005	F(1,14) = 0.062, *p* = 0.807, ηp^2^ = 0.004
PTH (pg/mL)	71.28 ± 36.11	69.06 ± 26.85	−2.22 (−3.10%), [95% CI: −10.92, 6.50]	71.13 ± 35.87	71.18 ± 35.85	+0.05 (+0.07%), [95% CI: −0.09, 0.19]	−0.15 (−0.21%) [95% CI: −25.90, 25.60]	+2.12 (+2.98%) [95% CI: −20.54, 24.78]	F(1,14) = 0.212, *p* = 0.652, ηp^2^ = 0.015	F(1,14) = 0.237, *p* = 0.634, ηp^2^ = 0.017	F(1,14) = 0.259, *p* = 0.618, ηp^2^ = 0.018
Phosphorus (mg/dL)	3.49 ± 0.58	3.63 ± 0.51	+0.14 (+4.00%), [95% CI: −0.02, 0.30]	3.48 ± 0.55	3.47 ± 0.54	−0.01 (−0.29%), [95% CI: −0.02, 0.00]	−0.01 (−0.29%) [95% CI: −0.41, 0.39]	−0.16 (−4.61%) [95% CI: −0.53, 0.21]	F(1,14) = 3.706, *p* = 0.075, ηp^2^ = 0.209	F(1,14) = 2.621, *p* = 0.128, ηp^2^ = 0.158	F(1,14) = 2.660, *p* = 0.125, ηp^2^ = 0.160
ALP (U/L)	54.00 ± 14.30	54.66 ± 14.20	+0.66 (+1.20%), [95% CI: −3.23, 3.75]	54.00 ± 14.73	54.06 ± 14.71	+0.06 (+0.11%), [95% CI: −0.23, 0.35]	0.00 (0.00%) [95% CI: −10.38, 10.38]	−0.60 (−1.11%) [95% CI: −10.94, 9.74]	F(1,14) = 0.513, *p* = 0.485, ηp^2^ = 0.035	F(1,14) = 0.847, *p* = 0.373, ηp^2^ = 0.057	F(1,14) = 0.920, *p* = 0.354, ηp^2^ = 0.062

Note: Data are expressed as mean ± SD; analysis of variance was expressed in both time, group and time × group as F[IV degrees of freedom (df), error df] = F-ratio, *p* = significant level, ηp^2^ = partial eta squared. 95% CI: 95% confidence interval (lower bound/upper bound); PLR: Prolactin; PTH: Parathyroid Hormone; ALP: Alkaline phosphatase.

**Table 4:** DEXA measurements of the participants at baseline and at the end of the study.

	Group A (n_A_ = 15)			Group B (n_B_ = 15)			Group A vs. B		Analysis of Variance, *p* Value, ηp^2^		
	Baseline	Follow-Up	Change Mean (%), [95% CI: Lower/Higher Bound]	Baseline	Follow-Up	Change Mean (%), [95% CI: Lower/Higher Bound]	Change Mean (%), [95% CI: Lower/Higher Bound]		Group	Time	Group × Time
							Pre	Post			
BMD lumbar spine (L1–L4) (g/cm^2^)	0.84 ± 0.09	0.85 ± 0.10	+0.01 (+1.19%), [95% CI: 0.00, 0.02]	0.83 ± 0.10	0.83 ± 0.11	≈0.00 (0.0%), [95% CI: −0.01, 0.00]	+0.01 (+1.20%) [95% CI: −0.05, 0.07]	−0.02 (−2.40%) [95% CI: −0.09, 0.05]	F(1,14) = 3.689, *p* = 0.075, ηp^2^ = 0.209	F(1,14) = 0.868, *p* = 0.367, ηp^2^ = 0.209	F(1,14) = 2.494, *p* = 0.137, ηp^2^ = 0.151
BMD total hip (left) (g/cm^2^)	0.74 ± 0.10	0.77 ± 0.11	+0.02 (+4.05%), [95% CI: 0.01, 0.03]	0.74 ± 0.11	0.73 ± 0.09	−0.01 (−1.35%), [95% CI: −0.02, 0.00]	0.00 (0.00%) [95% CI: −0.07, 0.07]	−0.04 (−5.50%) [95% CI: −0.11, 0.03]	F(1,14) = 3.457, *p* = 0.084, ηp^2^ = 0.198	F(1,14) = 1.759, *p* = 0.206, ηp^2^ = 0.112	F(1,14) = 10.717, *p* = 0.006 *, ηp^2^ = 0.434
BMD total hip (right/dominant) (g/cm^2^)	0.76 ± 0.08	0.81 ± 0.08	+0.05 (+6.57%), [95% CI: 0.03, 0.06]	0.75 ± 0.08	0.75 ± 0.07	≈ 0.00 (0.0%), [95% CI: −0.01, 0.00]	−0.01 (−1.30%) [95% CI: −0.06, 0.04]	−0.06 (−8.00% [95% CI: −0.11, −0.01]	F(1,14) = 14.057, *p* = 0.002 *, ηp^2^ = 0.501	F(1,14) = 32.834, *p* < 0.001 *, ηp^2^ = 0.701	F(1,14) = 36.974, *p* < 0.001 *, ηp^2^ = 0.725
Total spine Τ-score	−2.37 ± 0.97	−2.32 ± 1.06	+0.05 (+2.1%), [95% CI: −0.12, 0.22]	−2.30 ± 0.81	−2.34 ± 0.82	−0.05 (−1.7%), [95% CI: −0.08, −0.01]	+0.07 (+3.00%) [95% CI: −0.57, 0.71]	−0.02 (−0.90%) [95% CI: −0.69, 0.66]	F(1,14) = 1.711, *p* = 0.212, ηp^2^ = 0.109	F(1,14) = 2.373, *p* = 0.146, ηp^2^ = 0.145	F(1,14) = 2.400, *p* = 0.144, ηp^2^ = 0.146
Total hip (left) Τ-score	−1.84 ± 0.74	−1.47 ± 1.09	+0.37 (+20.1%), [95% CI: 0.01, 0.73]	−1.90 ± 0.70	−1.92 ± 0.69	−0.02 (−1.1%), [95% CI: −0.05, 0.01]	−0.06 (−3.20%) [95% CI: −0.57,0.71]	−0.45 (−23.40%) [95% CI: −1.10, 0.20]	F(1,14) = 5.598, *p* = 0.033 *, ηp^2^ = 0.286	F(1,14) = 3.430, *p* = 0.085, ηp^2^ = 0.197	F(1,14) = 4.387, *p* = 0.055, ηp^2^ = 0.239
Total hip (right) Τ-score	−1.90 ± 0.69	−1.44 ± 0.87	+0.46 (+24.2%), [95% CI: 0.26, 0.66]	−2.06 ± 0.78	−2.10 ± 0.79	−0.04 (−1.9%), [95% CI: −0.08, 0.01]	−0.16 (−7.80%) [95% CI: −0.68, 0.37]	−0.66 (−31.40%) [95% CI: −1.25, −0.06]	F(1,14) = 14.611, *p* = 0.002 *, ηp^2^ = 0.511	F(1,14) = 15.578, *p* = 0.001 *, ηp^2^ = 0.527	F(1,14) = 22.246, *p* < 0.001, ηp^2^ = 0.614

Note: Data are expressed as mean ± SD; analysis of variance was expressed in both time, group and time × group as F[IV degrees of freedom (df), error df] = F-ratio, *p* = significant level, ηp^2^ = partial eta squared. * *p* < 0.05 indicates statistical significance. 95% CI: 95% confidence interval (lower bound/upper bound). BMD: Bone Mineral Density.

## Data Availability

The original contributions presented in this study are included in the article. Further inquiries can be directed to the corresponding author.
